# Event and Comment

**Published:** 1920-01

**Authors:** 


### DENTAL REGISTER

THE MAGAZINE OF DENTISTRY

Vol. LXXIV JANUARY, 1920 No. 1

### EVENT AND COMMENT

**Nineteen Twenty?** As the new year approaches and we busy ourselves with recording the accomplishments of the past year, we naturally wonder what is in store for us, or rather, what hope we have for a more satisfactory nineteen-twenty record than we made last year. We all, of course, recognize the many failures of the years that are gone, full of rich opportunities, some of which we utilized to good advantage, but most of which we failed to see at all; or perhaps, we did not adequately estimate their values or their relations to our particular needs, which, of course, is not strange, since none of us can claim omniscience—neither have we the capacity to make the most of the chances that come our way. But as a profession, have we made advancement during the past year, and are we better prepared to serve the people more intelligently and faithfully this new year? To answer this question adequately would require long thought and much writing, but we can each one answer it for himself perhaps with a degree of judgment that can only be based upon his acquirement of the knowledge and art produced by the profession and the way he has made use of it in serving his patients to their advantage.

If we would take time to look over the accomplishments of the past year that have been helpful in our professional activities, we should be thankful perhaps to many persons for suggestive helps that have made for progress in the practice of our profession. Our patients, our professional confrères, our journal literature, and our own mistakes, as well as the mistakes of others, should have taught us many things of real value. We can not catalogue all the good things we have learned, much less remember them, because most of them we have assimilated so unconsciously that we really believe we discovered most of the things we have accumulated. And so we have grown, some more than others, but we have all accumulated some things worth while. We do not quite realize how we got them, and perhaps we may be ignorant of their possession, but it is almost certain that if we have been busy serving others and have kept a reasonable contact with the activities of our calling, we have assimilated much that should make us better servants and wiser perhaps as to the fundamental details upon which all our practical conceits and actions are based. If then for the most of us we develop because of our environment—like the good plant—it ought to be worth while not only that we put our minds in receptive order but that we organize our lives and doings in such manner that it will be easy for us to appropriate all the good things that pass our way. Of course, we don't recognize all the best things that come our way, and unfortunately the spurious is often most attractive. This compels us to cultivate wisdom that we may discriminate. Most of us get wisdom with years of experience, but would it not be helpful if we were willing to learn something from those who have been over the road before us, and before we have gone so far that we become so hopelessly entangled in the ruts of habitual routine, that we hesitate to venture into new fields, however commendable and inviting they appear? In dealing with so serious a matter as the health of others it is wise to be conservative, but we have learned during the past year that we do not always serve our patients best when we save all devitalized teeth. It may not be at all improbable that during the coming year we shall get better data for determining how far we are justified in carrying the practice of pulp extirpation and the treatment of alveolar infections.

This problem is not yet fully solved, and will not be unless a more extended study of the entire matter is made than seems now probable. Our research men are doing good work but their work must be supplemented by the careful clinical application of scientific findings to a large number of patients by accurate practitioners, who by carrying out the research methods in detailed clinical application will reach a practical confirmation for all scientifically obtained data on this subject.

What better purpose can both young and old practitioners have for the new year than to determine, each in his own sphere of practice, that he will not only study this problem with the greatest care but with the hope that he may be able to make some contribution of value to this subject. If a large number would do this there can be little question as to the ultimate result. At any rate all who engage seriously in such an effort will be better prepared to take up this branch of practice and we shall certainly have to record next year a more definite progress than is possible at this time. Organize study clubs with one or many members and select some vital topic for study and experimentation, and make progress.

**Bogus American Dental Diplomas in England** Two men offering to sell dental diplomas in England were sent to prison from the "Old Bailey" in London, December 7th. The men were convicted on evidence supplied by the state authorities of Michigan, as they were offering to sell diplomas granted by St. Luke's Hospital, of Niles, Michigan. There is not now and never has been an institution of this name located in the state of Michigan having authority to confer any kind of degrees. A statement of this fact was sent to

London and it caused the men who were not dentists to acknowledge their fraud and they were sent to prison. They apparently had done some business, as they claimed to have collected about $750. They had 500 more diplomas to sell. It is hoped that nothing more will ever be heard from this infamous concern.

